# Molecular Epidemiology and Genotyping of Infectious Bronchitis Virus and Avian Metapneumovirus in Backyard and Commercial Chickens in Jimma Zone, Southwestern Ethiopia

**DOI:** 10.3390/vetsci7040187

**Published:** 2020-11-25

**Authors:** Dechassa Tegegne, Yosef Deneke, Takele Sori, Mukarim Abdurahaman, Nigatu Kebede, Mattia Cecchinato, Giovanni Franzo

**Affiliations:** 1 School of Veterinary Medicine, Jimma University College of Agriculture and Veterinary Medicine, P.O. Box 307 Jimma, Ethiopia; dachassat@yahoo.com (D.T.); yosefdeneke@yahoo.com (Y.D.); takele_aga@yahoo.com (T.S.); mukevet@yahoo.com (M.A.); 2 Aklilu Lemma Institute of Pathobiology, Addis Ababa University, P.O. Box 1176 Addis Ababa, Ethiopia; nigatukebede@yahoo.com; 3 Department of Animal Medicine, Production and Health (MAPS), University of Padua, 35020 Legnaro (PD), Italy; mattia.cecchinato@unipd.it

**Keywords:** IBV, aMPV, 793B genotype, Ethiopia, molecular epidemiology

## Abstract

Poultry production plays a relevant role in the Ethiopian economy and represents a source of poverty alleviation for several social classes. Infectious diseases can therefore significantly impact the economy and welfare. Despite infectious bronchitis virus (IBV) and avian metapneumovirus (aMPV) being present, the knowledge of their epidemiology and impact is extremely limited. In the present work, a cross-sectional study based on 500 tracheal swabs collected from 50 intensive and backyard unvaccinated flocks of the Jimma Zone was performed to investigate the circulation of these viruses and molecularly characterize them. IBV and aMPV presence was tested by molecular assays, and genotyping was carried out on positive samples. Accordingly, 6% (95% CI 2.06% to 16.22%) and 8% (95% CI 3.15% to 18.84%) of flocks tested IBV and aMPV positive, respectively. Particularly, IBV 793B (GI-13) strains were detected in backyard flocks only, and identical or closely related sequences (*p*-distance <2%) were detected in distantly spaced flocks, suggesting relevant viral circulation. On the contrary, both backyard and intensive flocks were affected by aMPV subtype B. Potential epidemiological links associated to the importation of parental birds from foreign countries could be established. These results highlight non-negligible circulation of these viruses, warranting further epidemiological studies and the evaluation of control measure implementation.

## 1. Introduction

Infectious bronchitis (IB) is a worldwide-distributed, acute, highly contagious viral disease of poultry caused by infectious bronchitis virus (IBV) and is characterized by lesions in the respiratory, reproductive, and urogenital organs [1,2,3,4]. It infects chickens of all ages, but young chicks are more susceptible as resistance increases with age [5]. IBV causes major economic losses in the chicken industry because of poor performance, decreased egg production and quality, and mortality, which can be high in presence of nephropathogenic strains or when secondary infections occur [6]. IBV infection is estimated to account for the third-highest losses among all livestock diseases, following avian influenza and echinococcosis [7].

IBV belongs to the family *Coronaviridae*, order *Nidovirales*, genus *Gammacoronavirus*, species *Avian coronavirus* [6,8]. Its genome consists of 27.6 kb single-stranded positive-sense RNA molecules with the following genomic organization: 5′-untranslated region (UTR)-1a/1ab-S-3a-3b-E-M-5a-5b-N-3′- UTR. The spike (S), membrane (M), envelope (E), and nucleocapsid (N) are structural viral proteins, while the non-structural ones are encoded by two polyproteins (pp1a and pp1ab) [9]. Additionally, accessory proteins, 3a and 3b and 5a and 5b, have also been identified [10,11]. Among others, the spike protein (S) is widely studied because of its genetic variability and biological role.

The spike protein is cleaved into subunits S1 (amino-terminal component) and S2 (carboxy-terminal component) [9]. The S1 subunit is responsible for tropism, receptor attachment, neutralizing antibodies, and cell-mediated immune response induction, while S2 anchors S1 to the viral membrane [12]. After the first IBV serotype was identified [13] the virus was reported in different parts of USA [14,15] and a wide range of different IBV serotypes and genotypes were described worldwide [16,17,18]. The nomenclature and genotyping of IBV lacked consistency, mainly due to the rapid appearance of novel variants, unshared nomenclature criteria, and variable parts of S1 being sequenced to infer phylogenetic trees. However, a rational and standardized nomenclature of the IBV genetic groups was recently defined [19]. Accordingly, six genotypes comprising 32 lineages were recognized, although other unassigned recombinants with inter-lineage origin exist. In spite of worldwide efforts to control IBV so far, it remains a challenge for the poultry industry, mainly due to IBV’s extensive genetic diversity, short generation time, and high mutation rate [9,20]. The continuous emergence of genetic diversity and the selection process affect cross-protection among IBV variants, limiting the efficacy of natural or vaccine immunity [18,21].

The IBV distribution and diversity differ remarkably among genotypes [19]; for example, the Arkansas strain (GI-9) of IBV is the major strain in the USA [22] and has rarely been reported outside the USA. Similarly, D1466 (GII-1) is hardly ever isolated outside western Europe [23]. In contrast, the 793B (GI-13) and QX (GI-19) strains are widely distributed in Asia, Europe, and Africa, but have not been reported in the USA and Australia.

In Africa, IBV is one of the most important viral diseases that threaten chicken production [24]. So far, vaccine genotypes (Mass and 793B strains) and several other non-vaccine types have been reported [18], although specifically designed studies to define IBV (and relative lineages) prevalence have not been performed yet. IBV was first reported in Africa in 1950 in Egypt from birds with respiratory symptoms [25]. A number of IBV serotypes, antigenic variants, and field strains have since been isolated. To the best of the authors’ knowledge, only two studies have been conducted so far. Hutton et al. [26] reported the presence of the 793B genotype in commercial chicken farms with high (94.5%) seroprevalence, and sequencing revealed a 92–95% relatedness to the French isolate FR-94047-94 strain. Moreover, Tesfaye et al. reported M41, D-274, 793B, and QX serotypes, with IBV prevalence rates of 74.88% and 68.75% in unvaccinated backyard and commercial farms, respectively [27].

*Avian metapneumovirus* (aMPV) has been much less investigated. However, recent studies have indicated the increasing role of aMPV in respiratory diseases in broiler farms, mainly as a primarily pathogen [28]. *Avian Metapneumovirus* belongs to the family *Pneumoviridae* and genus *Metapneumovirus*, causing diseases like turkey rhinotracheitis in turkeys [29], swollen head syndrome in broilers and broiler breeders when associated with bacterial infections [30], and a decrease in egg production in layers and breeders [31].

*Avian metapneumovirus* contains a non-segmented, single-stranded, negative-sense RNA genome of approximately 13 kb in length, organized as 3′ leader N P M F M2 SH G L trailer 5′ [32]. After its first detection in South Africa in 1978 [33], analysis of attachment protein revealed the presence of four subtypes [34]. aMPV-A and B are widely distributed, while subtype C has been reported in only a few countries, including the USA, France, China, and South Korea [35,36], and subgroup D has only been reported in France [37].

IBV and aMPV’s epidemiology, strain diversity, molecular characterization, and regional distribution have not been comprehensively reported in Africa, particularly in Ethiopia [38].

However, there is no doubt among veterinarians about the economic importance of IBV and aMPV in the Ethiopian poultry industry. Therefore, the purpose of this study was to molecularly characterize aMPV and IBV in Jimma Zone and determine their genetic relationships with previously known strains using sequence data analysis.

## 2. Materials and Methods

### 2.1. The Study Area

Oromia Regional State is the largest state in Ethiopia, hosting nearly half of the country’s population and livestock, including poultry. The majority of the commercial poultry farms and breeding centers in Ethiopia are located in this region. Jimma is the capital city of Jimma Zone, which is located in Oromia Regional State. The total population of Jimma Zone is 2,642,114, of which 2,204,225 (88.66%) are members of rural communities engaged in agricultural activities for their livelihood. Jimma Zone is, therefore, a remarkable source of livestock which contribute to the country’s growth and domestic production: about 466,154 sheep, 194,677 goats, 1,718,284 cattle, 40,555 donkeys, 30,541 mules, 74,774 horses, and 1,774,116.36 chickens are kept in this area.

### 2.2. Study Design and Sampling Technique

A stratified cross-sectional study design was used in line with the primary objectives of this study. The sample size for pooled samples was calculated with the aim to estimate the infection prevalence with a confidence level of 95% and precision of 5%, assuming a conservative prevalence of 15%. Therefore, a minimum of 45 pooled samples was determined [39]. The sample size calculation was performed using EpiTools (https://epitools.ausvet.com.au/). Commercial poultry farms in Jimma city and backyard production systems from Jimma Zone were stratified according to production type, breed, and agro-ecological region.

From the selected poultry farms, 10 tracheal swabs were randomly collected, air-dried to prevent bacterial and mold growth, put in Eppendorf tubes, and transported to the Jimma University College of Agriculture and Veterinary Medicine (JUCAVM) Molecular Biotechnology Laboratory for storage at −80 °C. Tracheal swabs with dry ice were shipped to the Veterinary Infectious Disease Laboratory, Department of Animal Medicine, Production and Health, Padova University, Italy for PCR, sequencing, and genotyping. Whenever possible, the following data were collected: sampling date, farm geolocalization, animal category (broilers or layers), breed, age, presence of clinical signs, production (intensive and extensive) and housing system (backyard, commercial), population size, and vaccination used.

Overall, tracheal swabs were taken from a total of 500 chickens located in 50 farms (25 commercial farms with mean flock size 68 and age 9 months and 25 backyard premises with mean flock size 15 and age 4 months) during January and February 2020.

### 2.3. Data Analysis

Risk factors potentially associated to IBV and aMPV infection (i.e., age, breed of chicken, farm type, flock size) were assessed via logistic regression, conducted using SPSS version 20 software.

### 2.4. RNA Extraction, RT-PCR, and Sequencing

Individual samples were added to 500 µL of PBS and thoroughly vortexed. Then, 100 µL was collected from each of the 10 resuspended swabs collected in the same farm and pooled. The RNA was extracted from 200 µL of the obtained solution using the High Pure Viral RNA Kit (Roche, Basilea, Switzerland) following the manufacturer’s instructions. The pools were tested for IBV presence using the SuperScript™ III Platinum™ One-Step RT-PCR Kit (Thermo Fisher, Waltham, MA, USA), amplifying a 464 bp hyper-variable region of the S1 gene using the method described by Cavanagh et al. [40] with some modifications. Briefly, 5 µL of extracted RNA was added to a standard mix composed of 1X Reaction mix, 0.6 µM of XCE1+(CACTGGTAATTTTTCAGATGG) and XCE2 (CTCTATAAACACCCTTACA) primer pair, and 1 µL of SuperScript™ III RT/Platinum™Taq Mix (Thermo Fisher, Waltham, MA, USA). Molecular-biology-grade water was added up to a final volume of 25 µL. The selected thermal protocol was as follows: 50 °C for 30 min, then 95 °C for 2 min, followed by 45 cycles of 95 °C for 15 s, 50 °C for 20 s, and 68 °C for 40 s. A final extension step at 68 °C for 5 min was also performed. The positivity and specificity of the bands were verified by SYBRTMsafe (Thermo Fisher, Waltham, Massachusetts, USA) stained agarose gel electrophoresis, and amplicons were Sanger-sequenced in both directions using the same primers as for PCR at Macrogen Europe (Amsterdam, The Netherlands). The pools were also tested for aMPV presence using real-time RT-PCR as described by Cecchinato et al. [41], which allows the detection and differentiation of aMPV subtypes A and B.

### 2.5. Sequence Analysis

Chromatogram quality was evaluated using FinchTV (http://www.geospiza.com), and consensus sequences were assembled using ChromasPro (ChromasPro Version 2.0.0, Technelysium Pty Ltd., South Brisbane, Australia). The Ethiopian IBV sequences obtained in the present study were aligned with the reference strains obtained from Valastro et al. [19] using MAFFT [42]. Commonly applied vaccines strains were also included in the dataset. A phylogenetic tree was reconstructed using the maximum likelihood algorithm implemented in IQ-Tree [43], selecting as the substitution model the one with the lowest Akaike information criterion (AIC) value, calculated by the same program. The robustness of inferred clades was evaluated using the SH-like approximate likelihood ratio test (SH-aLRT) with 10,000 replicates. To evaluate the distribution of Ethiopian strains in the international scenario, an extensive dataset of IBV sequences of the corresponding genotype was downloaded from GenBank and included in the phylogenetic tree.

### 2.6. Ethical Approval and Consent

This study was approved by the Animal Research Ethics Review Committee of JUCAVM. Ethical clearance was obtained from the Ethics Committee of the College of Agriculture and Veterinary Medicine, Jimma University (ethical code R/GS /217/2012). Handling of the study animals throughout the study period was according to the World Organization for Animal Health (OIE) animal welfare guidelines.

## 3. Results

### Overview of IBV and aMPV Genotypes Detected

No significant effect of age (*P* = 0.436), chicken breed (*P* = 0.053), farm type (*P* = 0.086), or flock size (*P* = 0.257) was identified on IBV and aMPV infection frequency using linear regression. IBV-positive results were found in three backyard production systems only (Table 1), in local Horro breeds raised for meat and egg production, corresponding to 6% (95% CI 2.06–16.22%) farm-level prevalence, while the individual prevalence estimated from pooled samples was 0.6% (95% CI 0.01–1.75%). On the contrary, aMPV subtype B was detected in two intensive (Jimma city farm) and two backyard farms (Table 1), and in Bovans Brown and local Horro breeds (Asendabo and Setema), corresponding to 8% (95% CI 3.15–18.84%) farm-level prevalence, while the individual prevalence estimated from pooled samples was 1.04% (95% CI 0.34–2.43%).

**Figure 1:**
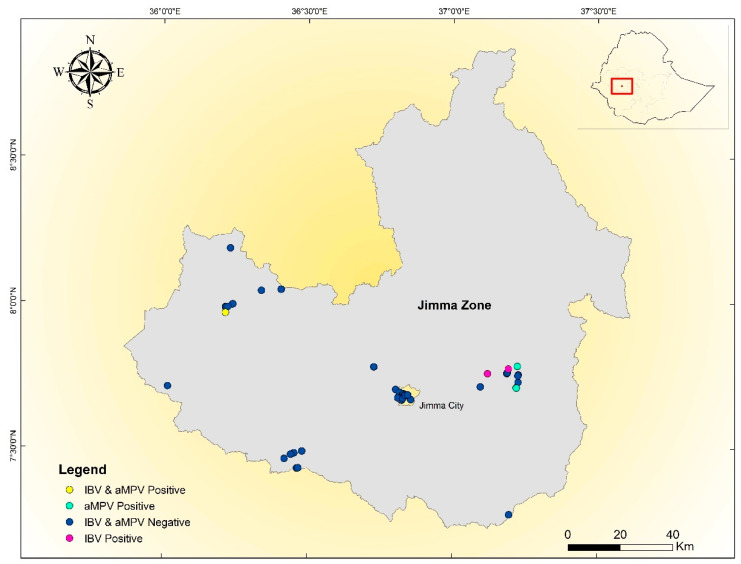
Farm locations of infectious bronchitis virus (IBV)-positive (pink), avian metapneumovirus (aMPV)-positive (green), co-infected (yellow), and negative (blue) cases.

The geographical distribution of the farms is reported in Figure 1 and Table 1. In five farms, different clinical signs were observed predominantly: respiratory symptoms (*n* (number chickens in the farm) = 19), enteric symptoms (*n* = 25), watery eyes or mucus in the nares (*n* = 15), lameness and crooked neck (*n* = 5), and blindness (*n* = 1). Clinical signs were not detected in the IBV-positive flocks; however, the intensive farms positive for aMPV showed respiratory signs. No documented data indicated either backyard or commercial farm vaccination against IBV or aMPV among the farms included in this study.

**Figure 2:**
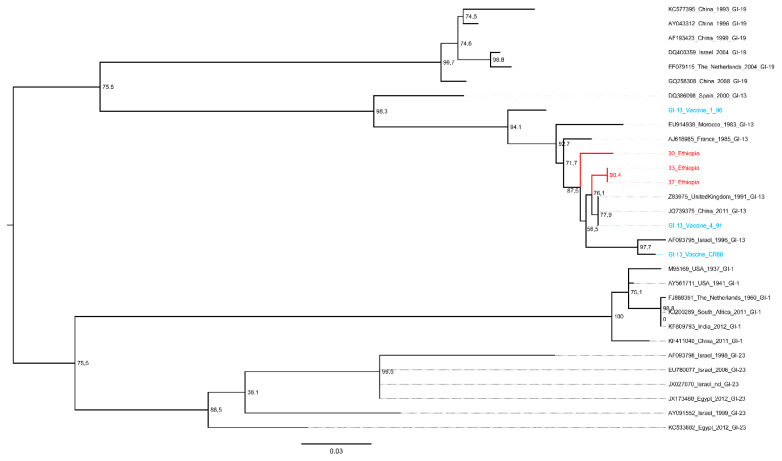
Maximum-likelihood phylogenetic tree comparing the IBV strains obtained in the present study (highlighted in red) with some reference strains proposed by Valastro et al., 2016. Reference vaccine strains are reported in blue.

Sequence analysis of the hypervariable region of the S1 gene (Figure 2) showed the circulation of 793B genotypes (GI-13) only, Strains 33 and 37 being 100% identical, while their percentage of identity with Strain 30 was 98%. The detected Ethiopian IBV strains were distantly related (minima of 4 and 9 mismatches) to the commonly used 793B (GI-13) live vaccines (i.e., 4/91, 1/96, and CR88) and were thus classified as likely field strains.

Comparison of the obtained sequences with the reference ones demonstrated a relatively high percentage of identity with the oldest known isolate of this genotype, EU914938-Morocco-1983 GI 13 (nucleotide identity of 95.4%), as well as with Z83975 United Kingdom 1991 GI-13 (nucleotide identity of 98–99%) and JQ739375|China-2011-GI-13 (nucleotide identity of 98–99%). However, when a broader 793B dataset was evaluated, a closer phylogenetic relationship was observed with strains collected in India and China (Supplementary Materials Figure S1).

## 4. Discussion

Especially in the small-scale village context, chicken farming can significantly contribute to poverty alleviation by means of income generation and household food security [44,45], and it represents a main source of self-reliance for women [46]. Poultry are kept by all levels of society, from the landless rural poor to the well-off in the cities. Among multiple factors that affect the poultry industry in Ethiopia, the major limiting ones are infectious diseases [47]. The most relevant poultry viral diseases in Ethiopia are Newcastle disease, infectious bursal disease, and Marek’s disease [48]. However, IBV accounts for the third-highest economic losses among all livestock diseases in the poultry industry worldwide by causing respiratory, reproductive, and renal damage, which leads to decreased productive performance and increased mortality [7,16]. Nevertheless, despite its enormous economic burden, it is underestimated and neglected in Ethiopia.

Different from the serological survey by Tesfaye et al., only genotype 793B (currently named GI-13, based on [19]) was found in the present study. However, it must be stressed that serological characterization is often challenging due to partial cross-reactivity or mutations in specific epitopes. Therefore, more extensive molecular epidemiology studies should be performed to investigate whether the herein-described scenario is representative of the overall Ethiopian one or different IBV genotypes demonstrate a different spatial or temporal pattern in this country.

The 793B genotype frequency in unvaccinated backyard farms was 6%, lower than in most previous studies—16.04% in Oman [49], 40.62% in Iraq [50], 38.4–41.3% in Iran [51,52], and 12.7% in Ivory Coast [53]—but greater than in one study conducted in Nigeria (2.9%) [54]. Using logistic regression analysis, no association between any of the examined risk factors and the detection of IBV in a flock was demonstrated at the set statistical significance level. However, the limited number of positive samples surely prevented the identification of statistical association. In fact, IBV-positive results were found in backyard production systems only, suggesting that IBV circulates more easily in this setting.

Although a low IBV infectious pressure cannot be excluded in Ethiopia, the high seroprevalence levels observed in previous studies seem to contradict this hypothesis. Many factors could have contributed to the low detection ratio, including the low animal/farm density and the old animal age. In fact, most animals were some months old, while IBV infection often occurs in early life phases. Therefore, previously acquired natural immunity could have decreased the infection frequency in the tested population. Younger animals could represent a future research target to investigate whether IBV infection is common in early life phases and to evaluate its impact and the potential usefulness of extensive vaccination protocol implementation.

The dominance of 793B is not surprising, considering its widespread use as a vaccine [55,56,57,58]. However, in the considered Ethiopian farms, no IBV vaccination was implemented. Therefore, the positive results were likely due to natural infection, as confirmed by the high genetic distance compared to commonly applied vaccines. Alternatively, the detected strains could be descended from vaccine strains that persistently circulated and evolved in the considered geographic area, acquiring a “field-strain behavior”.

The 793B strains detected within the backyard flocks shared a high nucleotide sequence identity (i.e., 98–100%), and the phylogenetic analysis based on the S1 hypervariable gene sequence demonstrated a certain clustering, indicating that closely related strains may be circulating between different, distantly located, Ethiopian backyard flocks and even regions (Figure 1).

In the absence of strict biosecurity measures, several spreading sources could be involved, including wild birds, wherein IBV-like viruses have been identified from time to time. This is particularly true in the case of backyard chickens that have access to the outdoors and live in close contact with wild birds [59,60]. Therefore, their role in viral spreading cannot be excluded and deserves further investigation.

Comparison of the S1 hypervariable gene sequence of Ethiopian 793B strains demonstrated a percentage of identity higher than 96% with most reference strains collected from Europe, including the ancient French isolate AJ618985_France-1985-GI 13, but especially with Asian strains. Parental chicken stocks were imported into Ethiopia beginning in the early 1950s [61] from different countries of Europe, Asia, and Africa to improve indigenous Ethiopian breeds and can thus be considered a likely introduction route into Ethiopia [62].

Although genetically distinct, the relatively close relatedness demonstrated on the basis of sequence analysis with commercial homologous vaccines might indicate a higher chance of good protection [18]. Therefore, commercial vaccines should confer protection, and their application deserves careful evaluation.

The results reported in this study evidenced the presence of aMPV in commercial and backyard farms. The viral load of aMPV was extremely low (cp of ~40) and no sequencing was possible. However, the use of specific probes allowed us to identify the aMPV-B subtype. aMPV-B was detected in both production systems (intensive and backyard) in animals raised for dual purposes (egg and meat production). Previously, subtype aMPV-B was reported in central Ethiopia in institutional farms [26]. In this study, aMPV was detected in both Bovans Brown breeds and local Horro breeds. Interestingly, the parent chicken stocks of the Bovans Brown breeds were imported into Ethiopia from Europe. The introduction of foreign strains could represent a possible route of viral entry for aMPV also, since the subtype B virus is by far the predominant one in Europe [30,63,64]. Other studies are currently ongoing to better characterize aMPV molecular epidemiology in Ethiopia and to understand its clinical and economic relevance.

The present study demonstrates the relevant circulation of two main poultry pathogens in Ethiopia and provides a molecular characterization of IBV strains. Since the old age of tested animals could have decreased infection prevalence and disease occurrence, the pathogens’ economic impact might be considered even more intense. Commercial vaccines against the reported genotypes and subtypes are widely available. This information, combined with previous study results, will be of help in the implementation of vaccination strategies directed toward the actually circulating genotypes, instead of generic ones. In the future, more focused studies should be performed to investigate the impact of these strategies and their potential benefits for the Ethiopian poultry sector’s productivity and overall economy and welfare.

## Figures and Tables

**Table 1:** Number of pooled samples collected, IBV and aMPV positive, classified according to sample location and production type.

Place	No. of Pooled Samples	Positive (n) Farms	
		IBV	aMPV
**Intensive**			
Jimma city and surroundings	16		2
Goma	4		
Shebe	5		
**Backyard**			
Setema	4		
Sigimo	3	1	2
Dedo	4		
Omonada	4		
Asendabo	5	2	
Dedo, Kersa	5	0	

## Supplementary Materials

The following are available online at https://www.mdpi.com/2306-7381/7/4/187/s1, Figure S1: Phylogenetic tree reconstructed based on a broad dataset of IBV strains. The Ethiopian sequences have been colored in red, while the corresponding clade is highlighted in yellow.
