# Supplementary figures and images for: Molecular Epidemiology and Genotyping of Infectious Bronchitis Virus and Avian Metapneumovirus in Backyard and Commercial Chickens in Jimma Zone, Southwestern Ethiopia
Source: Vet Sci. 2020 Nov 25;7(4):187. doi: 10.3390/vetsci7040187 (PMC7711717; doi:10.3390/vetsci7040187)

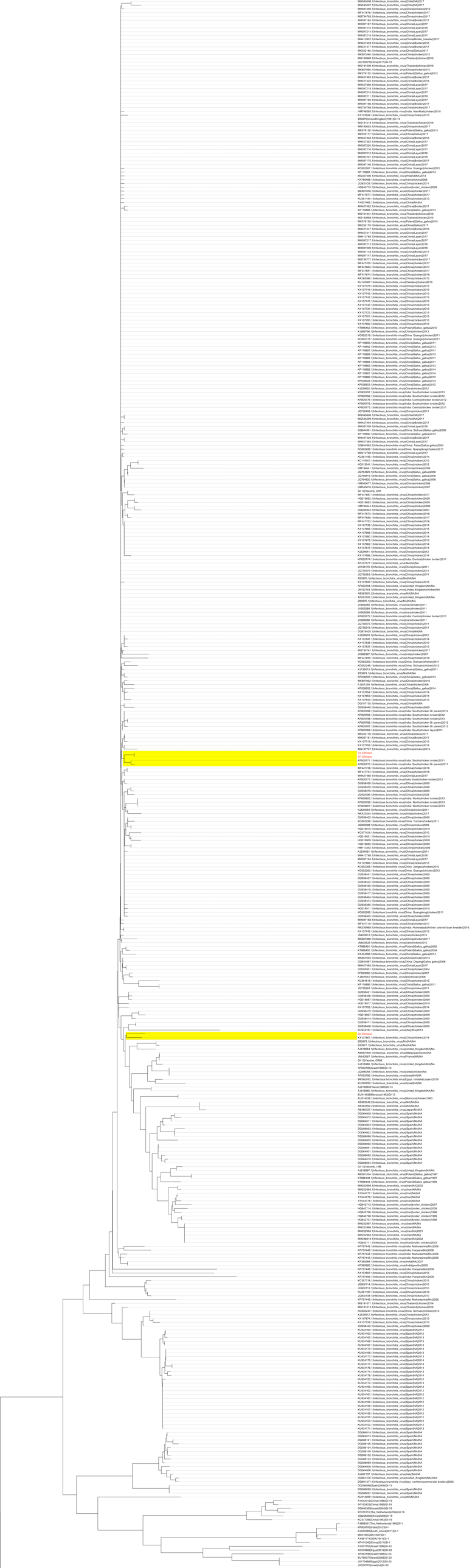

Supplement: Supplementary file 1 [file vetsci-07-00187-s001.pdf]
